# R5X4 HIV-1 coreceptor use in primary target cells: implications for coreceptor entry blocking strategies

**DOI:** 10.1186/1479-5876-9-S1-S3

**Published:** 2011-01-27

**Authors:** Lamorris M Loftin, Martha Kienzle, Yanjie Yi, Ronald G Collman

**Affiliations:** 1 Departments of Medicine and Microbiology, University of Pennsylvania School of Medicine, 522 Johnson Pavilion, 36th & Hamilton Walk, Philadelphia, PA 19104-6060, USA

## Abstract

Entry coreceptor use by HIV-1 plays a pivotal role in viral transmission, pathogenesis and disease progression.  In many HIV-1 infected individuals, there is an expansion in coreceptor use from CCR5 to include CXCR4, which is associated with accelerated disease progression.  While targeting HIV-1 envelope interactions with coreceptor during viral entry is an appealing approach to combat the virus, the methods of determining coreceptor use and the changes in coreceptor use that can occur during disease progression are important factors that may complicate the use of therapies targeting this stage of HIV-1 replication. Indicator cells are typically used to determine coreceptor use by HIV-1 *in vitro*, but the coreceptors used on these cells can differ from those used on primary cell targets.  V3 based genetic sequence algorithms are another method used to predict coreceptor use by HIV-1 strains.  However, these algorithms were developed to predict coreceptor use in cell lines and not primary cells and, furthermore, are not highly accurate for some classes of viruses.  This article focuses on R5X4 HIV-1, the earliest CXCR4-using variants, reviewing the pattern of coreceptor use on primary CD4+ lymphocytes and macrophages, the relationship between primary cell coreceptor use and the two principal approaches to coreceptor analysis (genetic prediction and indicator cell phenotyping), and the implications of primary cell coreceptor use by these strains for treatment with a new class of small molecule antagonists that inhibit CCR5-mediated entry.  These are important questions to consider given the development of new CCR5 blocking therapies and the prognosis associated with CXCR4 use.

## Introduction

HIV-1 isolates can be divided into three broad groups based on coreceptor use.  Variants with singular use of CCR5 or CXCR4 are termed R5 and X4 viruses, respectively, while those capable of using both coreceptors are termed R5X4 [1,2].  New infections are almost always established by R5 variants while the emergence of CXCR4-using viruses typically occurs later in a proportion of infected individuals and is associated with an accelerated decline in the number of peripheral blood CD4+ lymphocytes and a more rapid progression to AIDS and death [3-5]. R5X4 viruses are the first variants with CXCR4 use to emerge during viral evolution *in vivo *[6], so understanding coreceptor use on primary target cells by these viruses is critical to elucidating this aspect of pathogenesis and also has implications for the new line of therapeutics targeting viral entry.

CCR5 is expressed principally by memory CD4+ T cells and monocyte/macrophages, while CXCR4 is expressed by both naïve and memory CD4+ T cells and, at lower levels, by monocyte/macrophages [6,7].  Indicator cell lines that express CD4 in conjunction with CCR5 or CXCR4 are typically used to assess coreceptor use by HIV-1 [8,9].  While these cell lines are invaluable tools for studying certain aspects of HIV-1 entry and infection, we and others have shown coreceptor use on indicator cell lines may not accurately reflect coreceptor use on primary cells [2,10,11].  Here, we will discuss aspects of primary cell coreceptor use, focusing on areas where primary cell utilization and indicator cell use diverge, and in particular on R5X4 variants given their central role in viral evolution *in vivo*.

## R5 and X4 HIV-1 coreceptor use on macrophages

CD4+ macrophages and lymphocytes express CCR5 and CXCR4 [7,12], and efforts in the field have focused on defining the coreceptors used by HIV-1 on these cells and determining how viruses differ in their ability to use coreceptors on different primary cell targets including macrophages.

Early studies identified high concordance between the non-syncytia-inducing (NSI) phenotype and macrophage tropism [3], and the subsequent observation that NSI strains used CCR5 for entry but not CXCR4 [13-16] led to the expectation that R5 isolates would all likely replicate in macrophages.  Indeed, prototype R5 strains are typically highly macrophage-tropic, but this has not turned out to be the case for all R5 primary isolates as it was recently recognized that R5 strains can vary markedly in their ability to infect macrophages.

Interestingly, two different clinical patterns of R5 macrophage tropism have been described.  One set of data has reported that nearly all R5 Envs obtained directly from peripheral blood (i.e., without *in vitro* culture and selection) infect macrophages poorly whereas Envs from the central nervous system infect quite well [17,18]. CD4 levels on macrophages are quite low, and greater macrophage infection capacity among R5 strains has been linked to the ability to utilize CD4 at very low levels [17,18].  Importantly, CD4 binding plays a role in maintaining viral neutralization resistance by protecting the coreceptor binding site on Env, which is a potential target for neutralization but is only created after structural changes triggered by CD4 binding [19].  The immune privileged nature of the central nervous system is thought to allow emergence of such neutralization-sensitive, highly macrophage-tropic R5 variants [18,20,21].  In contrast, others have reported that R5 blood isolates from early stage infection infect macrophages poorly, but that as disease progresses, macrophage infection capacity increases [22], which is associated with an increasing ability to utilize lower levels of both CD4 and CCR5 by later stage variants [22,23].

In contrast to prototype R5 viruses, prototype X4 variants (which were isolated by serial passage in CD4+CXCR4+ transformed cell lines) are uniformly non-macrophage-tropic.  Subsequently, however, it has been recognized that macrophages do express CXCR4, albeit at low levels, and many X4 primary isolates are able to utilize macrophage CXCR4 even though prototypes cannot [24-27].  This phenotype among X4 variants is also linked, at least in part, to the ability of some X4 strains to use CXCR4 at the low levels expressed on macrophages, as CXCR4 overexpression can in some cases render macrophages permissive for infection by X4 prototypes [10].

## R5X4 HIV-1 coreceptor use on macrophages

**Figure 1:**
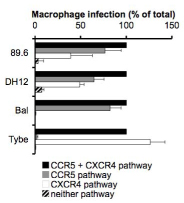
**R5X4 HIV-1 use CCR5 and CXCR4 on primary macrophages.** Monocyte-derived macrophages (MDM) were infected with HIV-1 luciferase-pseudotype viruses (5ng p24 Gag antigen) carrying representative prototype R5X4 envelope glycoproteins, along with control R5 (Bal) and X4 (Tybe) Env-containing viruses.  Infections were carried out without entry blocker or in the presence of the CCR5 antagonist Maraviroc (“CXCR4 pathway”; 5μM), CXCR4 antagonist AMD3100 (“CCR5 pathway”; 5μg/ml) or both inhibitors. Three days after infection, cells were lysed with 0.1% Triton, luciferase assay substrate (Promega) was added and luciferase activity (RLUs) was measured using a Dynex Revelation Luminometer.   Results represent normalized infection mediated by each coreceptor as a percentage of infection in the absence of antagonists and are means ± sem of infections done using cells from two different donors, each performed in triplicate.

Studies to determine which coreceptors R5X4 viruses use to infect primary macrophages have used replication competent and pseudotype viruses from different clades of HIV-1 [11,28].  Since macrophages express both coreceptors, unlike single coreceptor virus analysis, these studies have largely utilized small molecule antagonists to CCR5 or CXCR4 as a means of evaluating use of the unblocked coreceptor.  These studies have shown that in the presence of a CCR5 or CXCR4 antagonist, infection by R5X4 HIV-1 still occurs and infection by these viruses is fully blocked only when both antagonists are present.  The proportional contribution of each coreceptor to total infection of macrophages can be determined by comparing entry through that coreceptor to entry in the absence of antagonists.  As shown by the results from a representative group of R5X4 viruses in Fig 1 (and expanded upon for R5X4 isolates more broadly in ([11,28]), this analysis reveals that the level of viral entry that occurs through a single coreceptor is reduced relative to infection when both coreceptors are available.  Thus, both coreceptors make substantial contributions to the overall infection of macrophages by R5X4 HIV-1, although there are modest differences between isolates in the proportion of total entry mediated by each coreceptor.

## R5X4 HIV-1 coreceptor use on CD4+ lymphocytes

**Figure 2:**
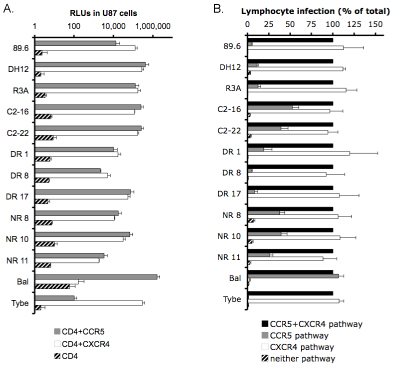
**Coreceptor use by R5X4 HIV-1 on indicator cell lines and primary CD4+ lymphocytes.** (A) Coreceptor use on U87 indicator cells by R5X4 HIV-1. U87 CD4, CD4/CCR5 or CD4/CXCR4 cells were infected with HIV-1 pseudotype viruses (5ng p24 Gag antigen). Three days after infection, cells were lysed and luciferase activity was measured.  Results are means  sem of two experiments performed in triplicate.  (B) Coreceptor-specific entry into CD4+ T lymphocytes.  Purified CD4+ lymphocytes were isolated by negative selection, stimulated with PHA for 3 days, infected with 5ng of HIV-1 pseudotype viruses in the absence or presence of coreceptor antagonists as described in Figure 1, maintained with IL-2 and lysed four days later for measurement of luciferase expression.  Results represent normalized infection through each coreceptor and are means +/- sem of infections done using cells from three different donors, each performed in duplicate.

R5X4 variants have the capacity to use both CCR5 and CXCR4 on macrophages and indicator cell lines (Figs 1 and 2A), but in contrast, the pattern of coreceptor use by R5X4 HIV-1 on CD4+ lymphocytes from peripheral blood is quite different from that seen on those two cell types.   Initial reports using a similar coreceptor blocking strategy and prototype strains showed that R5X4 viruses used CXCR4 on lymphocytes but CCR5 use was minimal, and lymphocyte CCR5 use by R5X4 isolates was markedly impaired relative to infection by R5 viruses [11].  Furthermore, unlike macrophages, infection mediated by CXCR4 alone was equivalent to infection when both coreceptors were present, suggesting no additional contribution of CCR5 in the presence of the CXCR4 pathway.  More recently, using an expanded panel of R5X4 Envs from diverse sources, we found that that some R5X4 viruses do possess the ability to use CCR5 for entry into CD4+ lymphocytes [29].   A range of CCR5 use was observed among these R5X4 strains, with CCR5 making virtually no contribution to infection by some strains while nearly half the total amount of entry could be mediated by CCR5 for other strains.  However, despite more robust CCR5 use by some clones, CXCR4 remained the predominant coreceptor used on CD4+ lymphocytes for all R5X4 viruses. CD4+ lymphocytes in blood are a mixture of different subsets, with CXCR4 expressed on a larger percentage of cells than CCR5 [12,30,31].  Consequently, CXCR4 is likely to be the predominate coreceptor used by the majority of R5X4 strains. Strikingly, there was consistently no difference between infection of unblocked CD4+ T lymphocytes and infection mediated by CXCR4 alone, confirming that even though both pathways can be used, when CXCR4 is available there is no additional contribution to infection made by CCR5 (Fig. 2B).

## Mechanisms that regulate R5X4 HIV-1 use of CCR5 on CD4+ lymphocytes

**Figure 3:**
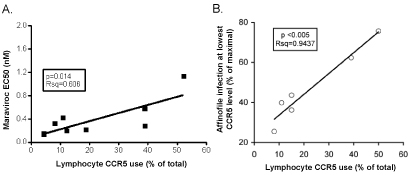
**CCR5 use on CD4+ lymphocytes by R5X4 HIV-1 correlates with CCR5 mediated entry efficiency.** (A) Relationship between R5X4 use of lymphocyte CCR5 and sensitivity to the CCR5 blocker Maraviroc.  The proportion of total entry into CD4+ lymphocytes that is mediated by CCR5 for each R5X4 virus is shown on the X axis while the Maraviroc EC50 determined using U87/CD4/CCR5 cells is shown on the Y axis.  Luciferase activity was measured as described in Figure 1, and EC50 values were determined using GraphPad Prism4 software.  (B) The correlation between R5X4 HIV-1 infection of Affinofile cells expressing CCR5 at low density and lymphocyte entry through CCR5.  CCR5/CD4-expressing Affinofile cells were induced to express varying levels of CCR5 at a constant level of CD4 and infected with R5X4 luciferase-expressing HIV-1 pseudotypes.  Infection of Affinofiles expressing low CCR5 levels was normalized to cell expressing CCR5 at maximal density and plotted on the Y axis against the proportion of total lymphocyte entry for each virus that is mediated by CCR5 on the X axis.

Differences in CCR5 use by R5X4 viruses on macrophages and lymphocytes imply the factors that regulate coreceptor use are cell-specific and differ on these primary cell types.  Furthermore, the fact that R5 strains uniformly use lymphocyte CCR5 efficiently indicates that there are virus-specific determinants as well.  In an attempt to identify the factors that regulate use of this coreceptor on CD4+ lymphocytes by R5X4 viruses [29], we found that greater ability to use CCR5 on primary lymphocytes correlated with reduced sensitivity to inhibition by the CCR5 antagonist Maraviroc and by another small molecule inhibitor M657 [32,33] (Fig 3 A and data not shown).  We also found a correlation between lymphocyte CCR5 use and resistance to blocking by anti-CCR5 monoclonal antibodies directed at the second extracellular loop of the protein (data not shown).  Since reduced sensitivity to CCR5 antagonists is often an indicator of greater efficiency of Env-CCR5 interactions, these results suggest that lymphocyte CCR5 use by R5X4 variants might be regulated by the efficiency of this interaction.

Therefore, the efficiency of CCR5 use was evaluated using an indicator cell line that allows independent manipulation of CD4 and CCR5 density [29].  At a physiologically relevant CD4 level (83,000 antibody binding sites (ABS)/cell, similar to the primary lymphocyte range of 63,000-100,000 ABS/cell [34]), there was a strong correlation between the ability of an R5X4 strain to use CCR5 to enter lymphocytes and relative infection of indicator cells at the lowest compared to the highest CCR5 densities (3,800 and 45,000 ABS/cell, respectively).  Thus, those R5X4 viruses that most effectively infected the indicator cells expressing minimal levels of CCR5 were also the variants that exhibited the greatest lymphocyte CCR5 use (Fig 3 B).  Since CCR5 is expressed at low density and on a small percentage of lymphocytes [30,34,35], these results indicate that viruses with the most efficient interactions with CCR5 are better able to scavenge the low levels of this coreceptor expressed on CD4+ lymphocytes to complete entry.  We confirmed this notion by upregulating CCR5 expression on CD4+ T lymphocytes through either lentivirus transduction or extended culture in IL-2, and found that for most R5X4 strains, CCR5 upregulation markedly enhanced their use of lymphocyte CCR5 for entry. Notably, the density of CCR5 on macrophages is much greater than on lymphocytes [34], and these findings suggest that the difference in CCR5 expression largely accounts for the disparate CCR5-mediated infection by R5X4 HIV-1 on these two primary cell types.

## CCR5 antagonists and R5X4 HIV-1

In recent years, a number of small molecule antagonists have been developed that target CCR5.  These agents are allosteric inhibitors that block infection by binding to the pocket created by the extracellular loops of CCR5, and cause conformational changes in the coreceptor that prevent its recognition and/or use by HIV-1 [36-38]. Maraviroc (Selzentry) [32] was the first CCR5 antagonist approved by the FDA for treatment-experienced patients and recently received approval for use in treatment-naïve patients.  Other coreceptor antagonists are under development, and a critical aspect of treatment with these agents is assessment of the presence of CXCR4-using viruses, which is a contraindication to treatment with CCR5 antagonists.   Therefore, assays for coreceptor use have received considerable attention.  The two approaches used to detect CXCR4 use are phenotyping of viral Envs in CCR5 or CXCR4-expressing indicator cells, and predictive algorithms based on sequences in the Env V3 domain, which is a principal determinant of coreceptor use [14,39,40].  Phenotyping of cloned plasma-derived Envs in indicator cells is presently utilized most frequently [41].

Both approaches have limitations, however.  Bulk phenotyping assays may not be sensitive enough to detect minor variants that use CXCR4 [42-44].  Furthermore, our studies of primary cells compared with cell lines show that indicator cell coreceptor use may not necessarily predict coreceptor use on primary cells.  On the other hand, while viral sequence algorithms are generally accurate at defining single coreceptor R5 and X4 variants or predicting certain qualities of these viruses, like the ability to induce syncytia formation, they frequently fail to identify R5X4 viruses.  In one study, a number of common sequence algorithms were used to predict the coreceptor use of viral strains that had been phenotyped as R5X4 on cell lines in vitro.  The sequence algorithms varied markedly in the prediction of coreceptor use, and failed to predict CXCR4 use for 10% to over 50% of the R5X4 clones analyzed, depending on the particular algorithm used for analysis [45].

**Figure 4:**
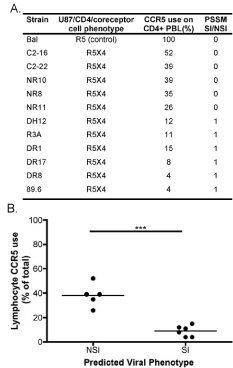
**R5X4 HIV-1 CCR5 use on CD4+ lymphocytes is associated with the predicted viral phenotype.** (A) Predicted NSI/SI phenotype of the R5X4 HIV-1 viruses.  V3 sequences from each virus were analyzed using the NSI/SI PSSM algorithm.  The table shows the SI or NSI prediction and coreceptor use on cell lines and primary lymphocytes for each R5X4 virus from Figures 1 and 2, respectively. The strains shown in the table are found in the following references: [63-68] (B) Mean CCR5 use on CD4+ lymphocytes by R5X4 HIV-1 grouped by predicted viral phenotype.  R5X4 viruses were grouped by SI or NSI phenotype from (A) with CCR5 use for each virus represented by black circles.  The mean lymphocyte CCR5 use for each group was calculated, and the means were compared using a two-tailed, unpaired t-test in GraphPad Prism 4 software (***p<0.0001).

A second consideration regarding sequence algorithms is that they have traditionally been used to predict coreceptor use by viral strains on indicator cell lines, but the success of these algorithms in predicting coreceptor use on primary cells is of central importance if they are to be used in clinical settings.  To this end, we determined the viral phenotypes predicted by a widely used position-specific scoring matrix (PSSM) algorithm [46] for a panel of R5X4 viruses for which coreceptor used had been determined on CD4+ lymphocytes (Fig 4).  This analysis showed that those R5X4 variants with significantly more efficient CCR5 use on primary CD4+ lymphocytes were likely to be incorrectly labeled as NSI, and thus erroneously presumed to be CCR5-restricted, by V3 sequence-based PSSM prediction.  In contrast, R5X4 strains that were restricted in their ability to use lymphocyte CCR5 were typically categorized as syncytium inducing (SI) by PSSM (Fig 4 A and 4 B).  Thus, using sequence algorithms, a number of R5X4 strains would be classified as R5 viruses, and this failure to detect CXCR4 could have important implications for entry blocking therapy as discussed below.

While phenotypic and sequence based approaches do not accurately determine coreceptor use by R5X4 strains on primary cells, it may be possible to modify these methods to increase their reliability.  As mentioned above, indicator cells used in phenotyping assays typically over-express CD4, CCR5 and CXCR4, which may contribute to the inaccuracy of this method in predicting coreceptor use by R5X4 strains on primary lymphocytes and macrophages.  The use of primary cells for routine viral phenotyping would be ideal, but this approach would be expensive, labor intensive and time consuming.  Alternatively, a system that closely mimics coreceptor expression on CD4+ lymphocytes and macrophages might be a more feasible option, and our results using Affinofile cells suggest that cell lines with receptor levels approximating those on primary cells may more accurately reflect coreceptor use on primary cells.  Thus, the development and utilization of cell lines that reflect the cellular landscape of primary targets may enhance the fidelity of in vitro viral phenotyping assays.

These data further suggest that it will be difficult to exclude R5X4 variants in patients considered for entry blocker therapy using sequence algorithms.  However, among viruses defined phenotypically as R5X4, the strong relationship between predictive algorithms and relative dependence on each pathway for primary lymphocyte entry indicate that sequences within Env regulate the efficiency of CCR5 use and, consequently, coreceptor use in the context of primary cells.  Identification of the specific elements within the R5X4 Env that regulate the efficiency of coreceptor use on primary cells would be necessary to improve the accuracy of these predictive algorithms before they could be used as the basis for entry therapy selection.

## Conclusions: R5X4 HIV-1 primary cell coreceptor use and implications for coreceptor blocking treatment

Addition of CCR5 antagonists to lymphocyte cultures *in vitro* does not block R5X4 replication [11], and a phase 2b study of Maraviroc safety and efficacy in patients infected with CXCR4-using viruses confirmed no change in plasma viral load between placebo and Maraviroc-treated groups [47].  CCR5 is expressed on a small percentage of peripheral blood CD4+ lymphocytes [12,30], however, many CCR5+ lymphocytes express CXCR4 as well [31].  Thus, when CCR5 is blocked, the CCR5+ subset of CD4+ lymphocytes can still be infected by R5X4 strains using CXCR4 for entry.  In clinical trials that attempted to exclude patients with CXCR4-using viruses, treatment with CCR5 blockers was generally successful [48,49].  However, some patients failed therapy, and while some strains acquired the ability to use antagonist-bound CCR5, a more common cause of treatment failure was the emergence of viruses able to use CXCR4 [42,49].  In this study and a similar trial with another small molecule antagonist, detectable CXCR4-using viruses appeared between screening and the start of therapy in some patients, while in other patients, the phenotypic screening assay failed to detect minor CXCR4-using variants [42,43].  In patients with pre-existing CXCR4-using viruses, suppression of the R5 variant may be closely tied to the rapid emergence of CXCR4-using strains, which can occur in less than 2 weeks in some patients [43,44].  R5X4 variants are typically the first CXCR4-using strains to emerge, so it is likely that these variants play a central role in emergence of populations that are insensitive to CCR5 entry blockers.  Since ~99% of plasma viremia is produced in CD4+ T cells [50-52], coreceptor selectivity in this primary target cell will be the principal determinant of plasma virus response to treatments.  One caveat is that CD4+ T cells in other compartments such as lymphoid tissues and gut mucosa are an important site of viral replication, and CCR5 is expressed on a higher proportion of CD4+ T cells in these compartments than in blood [53-55].  Thus, further studies will be needed to extend the characterization of coreceptor use by R5X4 variants to cells from these compartments.

In contrast, one would anticipate that a drug regimen that includes CCR5 blockers should reduce infection of macrophages by R5X4 HIV-1, since infection of these cells mediated by CXCR4 alone is typically less than that when both pathways are available.  The relative coreceptor preference on macrophages can differ between R5X4 strains, and consequently, the extent of reduced infection would depend on how effectively the R5X4 strain used macrophage CXCR4.  Treatment with CCR5 antagonists may also impact how R5X4 viruses use CXCR4 on macrophages.  In the absence of entry blocking therapy, it has been reported that *in vitro* sensitivity to CXCR4 antagonist decreases as disease progresses, suggesting CXCR4 use becomes more efficient at later stages of disease [56].  One might speculate whether inhibiting CCR5 use on dual coreceptor-expressing macrophages may create pressure that accelerates the evolution of more efficient CXCR4 use.

Reduced infection of macrophages by R5X4 HIV-1 may also impact other specific aspects of HIV-1 disease.  Neurological complications of HIV-1 infection are a result of damage induced by the release of neurotoxic factors from infected macrophages in the brain [57,58].  While most variants in the central nervous system are R5, R5X4 viruses have been found in this compartment, although less frequently than R5 strains [59].  The limited information available suggests that CCR5 blockers penetrate the central nervous system (CNS) at concentrations considerably lower than that in plasma [60].  Thus, on one hand, even at the lower concentrations found in the CNS, CCR5 antagonists may be somewhat effective in suppressing replication by R5 variants in macrophages found in this compartment.  Alternatively, incomplete suppression of macrophage-dependent replication in the brain might enable emergence or expansion of insensitive variants, particularly if R5X4 species are present.  Macrophages are also thought to be a source of HIV-1 in other tissues [61,62], where CCR5 blocking therapy might reduce macrophage-supported replication.

Antiretroviral therapy has made tremendous strides in recent years, and coreceptor entry blocking makes an important and unique contribution to the armamentarium of anti-HIV therapies.  However, unlike agents targeting other steps in the replication cycle, coreceptor blockers carry unique considerations and greater context-dependent variation due both to differences in target cell coreceptor expression and viral coreceptor utilization.  While exclusion of patients with certain types of virus may be possible, it is sometimes difficult to identify dual coreceptor-using variants.  Coreceptor use by R5X4 HIV-1 differs on primary macrophages and lymphocytes; consequently, the impact of entry blocking therapy may differ as well.  Thus, it is important to continue defining the role of coreceptor use in primary target cells and the consequences for pathogenesis, particularly by R5X4 viruses, in order to understand how new entry blocking agents may impact disease.

## List of abbreviations used

MDM: Monocyte derived macrophage; CCR5: C-C chemokine receptor 5; CXCR4: C-X-C chemokine receptor 4; PSSM: Position Specific Scoring Matrices.

## Competing interests

The authors declare no competing financial or other interests.
